# Transcriptome Analysis Reveals the Role of Cellular Calcium Disorder in Varicella Zoster Virus-Induced Post-Herpetic Neuralgia

**DOI:** 10.3389/fnmol.2021.665931

**Published:** 2021-05-17

**Authors:** Songbin Wu, Shaomin Yang, Mingxi Ou, Jiamin Chen, Jiabing Huang, Donglin Xiong, Wuping Sun, Lizu Xiao

**Affiliations:** ^1^Shenzhen Municipal Key Laboratory for Pain Medicine, Department of Pain Medicine, Shenzhen Nanshan People’s Hospital, The 6th Affiliated Hospital of Shenzhen University Health Science Center, Shenzhen, China; ^2^Department of Chemistry, University of Science and Technology of China, Hefei, China; ^3^Vanke Bilingual School (VBS), Shenzhen, China

**Keywords:** post-herpetic neuralgia, RNA-seq, VZV, calcium-related genes, calcium channel, Ca^2+^

## Abstract

As a typical neuropathic pain, post-herpetic neuralgia (PHN) is a common complication of herpes zoster (HZ), which seriously affects the normal life and work of patients. The unclear pathogenesis and lack of effective drugs make the clinical efficacy of PHN unsatisfactory. Here, we obtained the transcriptome profile of neuroblastoma cells (SH-SY5Y) and DRG in rats infected with varicella zoster virus (VZV) by transcriptome sequencing (RNA-Seq) combined with publicly available gene array data sets. Next, the data processing of the transcriptome map was analyzed using bioinformatics methods, including the screening of differentially expressed genes (DEGs), Gene Ontology (GO), and the Kyoto Encyclopedia of Genes and Genomes (KEGG) analysis. Finally, real-time fluorescent quantitative PCR (qRT-PCR) was used to detect the expression of calcium-related genes, and calcium fluorescent probes and calcium colorimetry were used to evaluate the distribution and content of calcium ions in cells after VZV infection. Transcriptome data analysis (GO and KEGG enrichment analysis) showed that calcium disorder played an important role in SH-SY5Y cells infected by VZV and dorsal root ganglion (DRG) of the PHN rat model. The results of qRT-PCR showed that the expression levels of calcium-related genes *BHLHA15*, *CACNA1F*, *CACNG1*, *CHRNA9*, and *STC2* were significantly upregulated, while the expression levels of *CHRNA10*, *HRC*, and *TNNT3* were significantly downregulated in SH-SY5Y cells infected with VZV. Our calcium fluorescent probe and calcium colorimetric test results showed that VZV could change the distribution of calcium ions in infected cells and significantly increase the intracellular calcium content. In conclusion, our results revealed that the persistence of calcium disorder caused by VZV in nerve cells might be a crucial cause of herpetic neuralgia, and a potential target for clinical diagnosis and treatment of PHN.

## Introduction

As a member of the alphaherpesvirinae subfamily, varicella zoster virus (VZV) is a common human pathogen that causes chickenpox during the initial infection, and reactivation from latently infected sensory neurons can cause herpes zoster (HZ) (Oliver et al., 2017; Yang et al., 2017). As a common complication of HZ, neuropathic pain is generally considered to be associated with neuronal damage and inflammation caused by reactivated VZV (Gilden et al., 2000; Gnann and Whitley, 2002). The outbreak of HZ rash usually precedes neuropathic pain, and after the rash is cured, neuropathic pain and discomfort such as allodynia and itching may develop further, eventually resulting in “post-herpetic neuralgia” (PHN) (Oxman et al., 2008). Herpetic neuralgia is usually defined as persistent pain and often accompanied by burning and hyperalgesia, a type of severe pain that can be triggered by touching or rubbing the affected area (O’Connor and Paauw, 2013; Silva et al., 2017). Although the use of anti-herpes drugs (such as acyclovir and famciclovir) in the early stage of HZ is conducive to shortening the duration of skin lesions and reducing the complications associated with HZ to a certain extent, the pain is unable to be completely cured (Li et al., 2009; Field and Vere Hodge, 2013). In clinics, antidepressants, non-steroidal anti-inflammatory drugs (NSAIDs), and sympathetic nerve blockers are also commonly used to relieve herpetic neuralgia, but these treatments often fail to prevent the development of PHN (Gan et al., 2013). Besides, the HZ virus vaccines cannot completely eliminate the occurrence of PHN, although it achieves some success in preventing the occurrence of shingles and herpetic neuralgia (Walker et al., 2018; Lang and Aspinall, 2019). All in all, the lack of precise pathological mechanism and effective drugs makes the clinical treatment of PHN unsatisfactory.

Central nervous system diseases, including neuropathic pain, are usually associated with abnormal neuronal calcium homeostasis and calcium signaling (Hagenston and Simonetti, 2014). More and more evidence has shown that second messenger calcium and calcium-dependent pathways played an important role in central sensitization (Luo et al., 2008). In fact, several reports have confirmed that abnormal calcium channel physiology and expression are not only associated with neuropathic pain and diabetic neuralgia, but also chronic inflammatory pain and bone cancer pain (Fossat et al., 2010; Naziroglu et al., 2012; Bourinet et al., 2014; Hagenston and Simonetti, 2014; Liu et al., 2018). However, there are many significant differences in the underlying mechanisms of different pain patterns, and there is also diversity in calcium regulation changes in pain models caused by specific injury patterns or diseases (Xu and Yaksh, 2011; Hagenston and Simonetti, 2014). These changes in calcium regulation usually cause the accumulation of calcium ions in cells, and eventually lead to disturbances in the calcium signal activity triggered by synapses (Raymond and Redman, 2006; Hagenston and Bading, 2011; Bading, 2013). Furthermore, calcium signals have been confirmed to be involved in pain signal transduction of cells and functional plasticity changes, ultimately affecting the occurrence and persistence of pain (Hagenston and Simonetti, 2014). However, there is no report about calcium signals in herpetic neuralgia, and the role of calcium signals in the occurrence and development of PHN is still unclear.

Here, we obtained the transcriptome map of VZV-infected neuroblastoma (SH-SY5Y) cells through *in vitro* experiments. By Gene Ontology (GO) enrichment and Kyoto Encyclopedia of Genes and Genomes (KEGG) analysis of the differentially expressed genes (DEGs) produced by VZV infection of SH-SY5Y cells, we have obtained preliminary evidence that calcium signals are involved in nerve cell response to VZV. Next, we combined with the reported gene chip data of DRGs in the rat model of herpetic neuralgia induced by VZV, and further confirmed the important role of calcium signals in the rat model of herpetic neuralgia. Additionally, the VZV-infected SH-SY5Y cell transcriptome profile and the VZV-induced herpetic neuralgia rat DRG gene chip data were compared to obtain 52 identical DEGs. The enrichment results indicated that these identical DEGs were mainly related to the regulation of calcium signals. Finally, qRT-PCR, calcium fluorescence probe, and calcium colorimetric assay were used to reveal the direct evidence of VZV induced intracellular calcium disorder. Therefore, we speculated that the disturbance of intracellular calcium signals caused by VZV might be related to the occurrence of PHN and the maintenance of persistent pain.

## Materials and Methods

### Ethics Statement

This study was approved by the Ethics Committee of Shenzhen Nanshan People’s Hospital and The 6th Affiliated Hospital of Shenzhen University Health Science Center.

### Cells and Cell Culture

SH-SY5Y (ATCC, CRL-2266) and ARPE-19 (ATCC, CRL-2302) cells were cultured in Dulbecco’s Modified Eagle medium (DMEM) with 10% fetal bovine serum (FBS) and penicillin-streptomycin (100 U/ml and 100 μg/ml), all from Gibco/Life Technology. All cells were cultured at 37°C in a 5% CO_2_ atmosphere.

### Viruses and Infection

Varicella zoster virus recombinant Oka strain carrying GFP reporter gene (rOka-GFP), kindly provided by Tong Cheng (Development in Infectious Diseases, Xiamen University, Xiamen, China), propagated in ARPE-19 cells to generate cell-associated progeny virus. VZV has a distinctive cell-associated nature, and the virus inoculants were prepared from VZV-infected ARPE-19 monolayer cells with marked and equivalent-appearing cytopathic effect (CPE) reaching >80% of cells. As previously reported, a cell-free virus was produced from ARPE-19 cells infected with VZV. Briefly, ARPE-19 cells were infected with the virus and the supernatant and cell pellets were collected when about 80% of the cells developed severe CPEs (Jiang et al., 2017). The cell pellets collected from three T-175 bottles were resuspended in 5 ml of DMEM. The virus particles were released from the cells by ultrasonic treatment (noise isolating chamber; 20 kHz, 45% amplitude, 15 s) and centrifuged at low speed (Beckman microfuge 20R; 1,000 × *g*, 5 min, 4°C) to remove cell debris. The supernatant obtained from cell lysis was combined with cell culture supernatant and centrifuged at high speed (Beckman rotor sw32; 80,000 × *g*, 3 h, 4°C) to concentrate the virus. The centrifuged virus precipitate was resuspended in 200 μl DMEM, and the viral titer was determined by plaque formation in ARPE-19 cells. All the virus experiments were carried out in a biosafety laboratory under appropriate ethical and safety approval. The study was performed according to the guidelines of the competent national authority.

### Total RNA Extraction

Before virus infection, SH-SY5Y (8 × 10^5^ cells/ml) and ARPE-19 (8 × 10^5^ cells) were seeded in a six-well cell plate at an initial seeding density of 80% of the cell-attached area. The next day, cells were infected with 1 × 10^4^ plaque-forming units (PFU)/ml cell-free VZV. The cells were digested with trypsin and harvested at 1 × 10^3^ rpm after 24 and 48 h of virus infection, respectively. The cell pellet harvested by centrifugation was treated with TRIzol Reagent (Invitrogen, United States) according to the manufacturer’s manual. RNA precipitation was dissolved in RNase-free water (Thermo Fisher Scientific, United States), and RNA samples were treated with DNase (TaKaRa, Japan). All nucleic acids were quantified on a nanodrop spectrophotometer (Thermo Fisher Scientific, United States), and the OD-(260)/OD-(280) ratio of the total RNA was between 1.8 and 2.0. The total RNA of the samples meeting the above criteria was stored in a freezer at −80°C until use.

### RNA Sequence and Data Analysis

SH-SY5Y cells were infected with 1 × 10^4^ PFU/ml cell-free VZV-rOka for 24 h and 48 h, respectively. The cells of each group were harvested and dissolved in TRIzol Reagent (*n* = 3 per group). The mRNA library construction and transcriptome sequencing of each sample was completed by Beijing Genomics Institute (BGI, Shenzhen, China). BGISEQ-500 high-throughput sequencing platform was used to pair sequence the cDNA of the RNA fragment, and 6 G average raw data of each sample were obtained. SOAPnuke (v1.5.2) was used to filter the raw data of sequencing, including removed adapter contamination, low-quality base ratio, and unknown base (“N” base); afterward, clean reads were obtained and stored in FASTQ format (Li et al., 2008). The clean reads were mapped to the human genome (hg19) using HISAT2 (v2.0.4) with *Q*-value ≤ 0.05 (Kim et al., 2015). Bowtie2 (v2.2.5) was applied to align the clean reads to the reference coding gene set; then, the expression level of the gene was calculated by RSEM (v1.2.12) (Li and Dewey, 2011; Langmead and Salzberg, 2012). According to the quantitative results of gene expression, we screened DEGs among samples based on DEseq2 and edgeR algorithm (Robinson et al., 2010; Love et al., 2014). The gplots R package was used to construct the heatmaps^1^, and Draw Venn Diagram online tool was used to generate the Venn diagram^2^. Functional classification of DEGs between groups was performed using the DAVID 6.8^3^ (Huang et al., 2009) and KOBAS 3.0^4^ (Xie et al., 2011; Shen et al., 2019) online database. The sequencing data set supporting the results of this article has been submitted to the NCBI Gene Expression Omnibus (GEO) database, and the accession number is GSE141932. The transcriptome data set of dorsal root ganglion (DRG) in a rat model of PHN induced by VZV was obtained from GEO, and the access number is GSE64345.

### qRT-PCR for mRNA Quantification

Total RNA was extracted by using TRIzol Reagent (Invitrogen, United States), followed by treatment with 10 U of DNase (TaKaRa, Japan). The synthesis of cDNA from 500 ng of total RNA was reverse transcribed using SuperScript^®^ III CellsDirect cDNA Synthesis Kit (Invitrogen, United States). The primers were designed using Primer-BLAST from the National Center for Biotechnology Information (NCBI) (Table 1). Gene expression quantification was determined by qRT-PCR using Fast SYBR^TM^ Green Master Mix (Applied Biosystems, United States) following the manufacturer’s instructions. Data were collected and analyzed using ABI-7500 software (Applied Biosystems, United States). The results were normalized to a housekeeping gene (*GAPDH*) and relative expression shown as 2^–ΔΔCt^.

**TABLE 1:** Sequences of the primer for quantitative real-time RT-PCR.

Gene name	Forward primer (5′–3′)	Reverse primer (5′–3′)
*ORF61*	ACATCCCTGCGTTGTCTTT	TTGAGGTGGTTTCTGGTCTTA
*GAPDH*	CTGGGCTACACTGAGCACC	AAGTGGTCGTTGAGGGCAATG
*BHLHA15*	CGGATGCACAAGCTAAATAACG	GCCGTCAGCGATTTGATGTAG
*CACNA1F*	GGAAGCCCTTCGACATCCTC	GTAGGCCACGATCTTGAGCAC
*CACNG1*	GACAGCCGTGGTAACCGAC	GCTTGGTACAAATCCGCCAGA
*CHRNA9*	AAATCTGGCACGATGCCTATC	GCAGGACCACATTGGTGTTCA
*CHRNA10*	CAGATGCCTACCTACGATGGG	GGGAAGGCTGCTACATCCA
*HRC*	AGAGAATGGGCATCATTTCTGG	TCATCTCCGACTTTGTGGTCTT
*STC2*	GCGTGCAGGTTCAGTGTGA	GGCCAGTCTCCCTACTGCT
*TNNT3*	AGGAGCTGGTCGCTCTCAA	CCTTCTCTGCACGAATCCTCT

### Western Blot Analysis

ARPE-19 and SH-SY5Y cells were treated with 1 × 10^4^ PFU/ml of cell-free VZV for 48 h. Using Western and IP cell lysate, we successfully extracted the total cellular protein, and then a 10% sodium dodecyl sulfate-polyacrylamide (SDS-PAGE) gel was used to separate the proteins. The protein was then transferred onto the PVDF membrane (Millipore, United States), which would later be blocked with 5% non-fat dry milk (Bio-Rad, United States) dissolved in TBS-T for 1 h at room temperature. The antibodies were then incubated at 4°C overnight: anti-VZV gE antibody (ab272686) (Abcam, United States; 1:2000) and anti-β-Actin (#4970) (Cell Signaling Technology, United States; 1:2000). The next day, after TBS-T washing three times, HRP-conjugated secondary antibodies (ProteinTech, United States, 1:5000) were performed to be combined with the primary antibodies. Finally, the immunoblots were visualized by using an ECL substrate kit (Millipore, United States).

### Assessment of Intracellular Ca^2+^

The content of Ca^2+^ in the cells was measured by fluorescent Ca^2+^ indicator Rhod-2 AM (#40776ES50, Yeasen, China). According to the manufacturer’s manual, ARPE-19 cells were infected with VZV for 72 h, and the medium was removed and the cells were rinsed with 1 × PBS at room temperature three times. After staining with 4 μM Rhod-2 AM for 30 min at room temperature (25°C), the fluorescent indicator was removed. The cells were rinsed with 1 × PBS at room temperature three times before observation and then placed in a 37°C, 5% carbon dioxide humidified incubator for 30 min. The images were taken by the laser confocal microscope (Olympus FV3000, Japan) at the excitation wavelength of 549/578 nm.

### Calcium Assay

According to the manufacturer’s calcium colorimetric assay kit (# S1063S, Beyotime Biotechnology, China) operating manual, we determined the calcium ion content of the VZV-infected ARPE-19 and SH-SY5Y cell lysate and culture supernatant. After VZV infects ARPE-19 and SH-SY5Y cells, the cell culture medium is collected and rinsed with pre-cooled 1 × PBS two to three times. Add 200 μl of pre-cooled sample lysate to each sample to make the sample lysate fully contact the cells. After the cells are fully lysed, collect the cell lysate at 4°C, centrifuge at 12,000 × *g* for 5 min, and aspirate the supernatant. The standard curve of calcium ion content was obtained by calcium standard solution and chromogenic solution, and the absorbance value of each sample was quantified as the corresponding calcium ion concentration.

### Statistical Analysis

All quantitative data were presented as mean ± standard deviations (SD) from three or more independent experiments. The quantitative data were analyzed by one-way analysis of variance (ANOVA), and the statistical significance was analyzed by GraphPad Prism 8 (GraphPad Software, La Jolla, CA, United States). Fisher’s exact test was used to assess the differential expression of genes (Robinson and Oshlack, 2010). For the screening of DEGs, the genes with *q*-values lower than 0.05 and with a fold change ≥2 are considered to differentially expressed. Data were considered significant if *P* < 0.05 (^∗^), *P* < 0.01 (^∗∗^), or *P* < 0.001 (^∗∗∗^).

## Results

### ARPE-19 and SH-SY5Y Cells Infected With VZV

**FIGURE 1:**
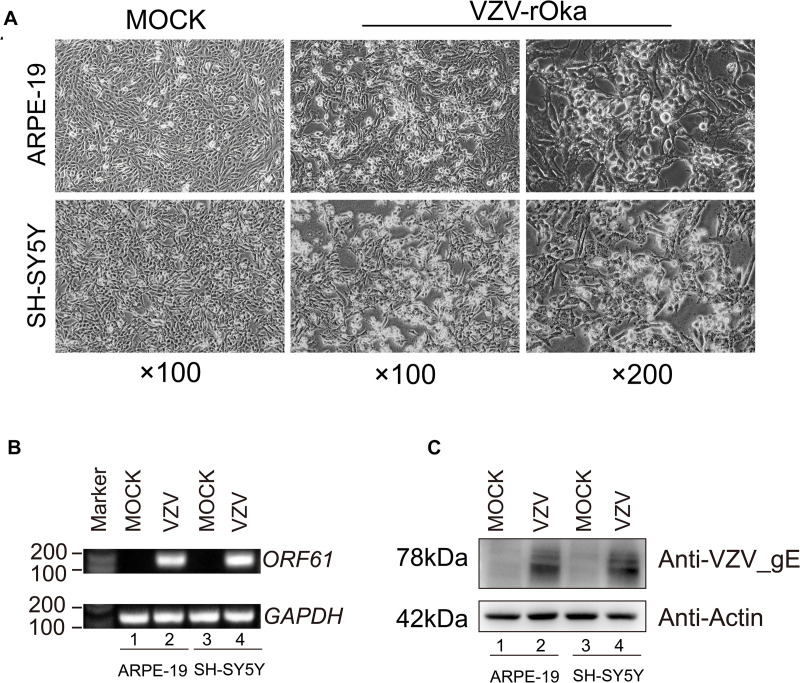
ARPE-19 and SH-SY5Y cells infected with VZV induced obvious cytopathic changes. **(A)** ARPE-19 and SH-SY5Y cells infected with VZV for 48 h induce obvious cytopathic changes, and cells not infected with (MOCK) virus maintain normal morphology and growth. **(B)** Real-time quantitative PCR was used to measure the expression of *ORF61* encoding the early phosphorylation protein of virus in ARPE-19 cells and SH-SY5Y cells after 48 h of VZV infection. **(C)** Western blot was used to evaluate the expression of VZV glycoprotein gE in ARPE-19 cells and SH-SY5Y cells.

Virus infection has strict species specificity, usually requiring to be parasitized in specific types of cells to meet the needs of virus replication. As previously reported, ARPE-19 cells and SH-SY5Y cells showed obvious cytopathic states such as roundness, swelling, and syncytial formation after 48 h of VZV infection compared with normal cells (MOCK group without virus infection) (Figure 1A; Jiang et al., 2017; Shakya et al., 2019). To further verify the successful infection of VZV, we measured the expression of *ORF61* gene encoding the virus early phosphorylation protein by qRT-PCR and evaluated the expression of VZV membrane glycoprotein E (gE) by Western blot. As shown in Figures 1B,C, the expression of *ORF61* gene and gE protein was observed in ARPE-19 cells and SH-SY5Y cells after 48 h of VZV infection, while no corresponding bands were observed in mock group cells. In conclusion, our results showed that VZV successfully infected ARPE-19 cells and SH-SY5Y cells and expressed viral genes and proteins in infected cells.

### Gene Ontology Analysis of DEGs in SH-SY5Y Cells Infected With VZV

**FIGURE 2:**
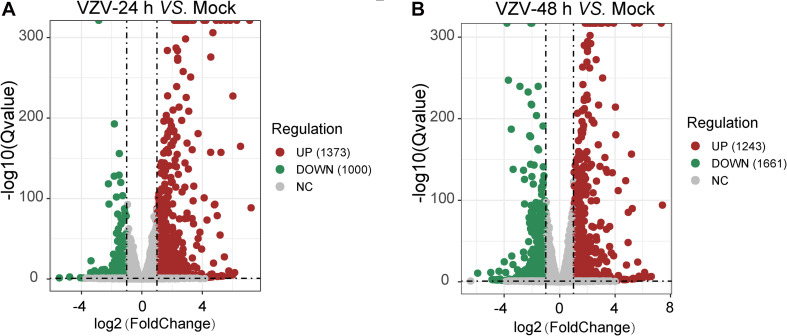
A mass number of DEGs were induced by VZV in SH-SY5Y cells. **(A)** Volcanic map showed the number and distribution of DEGs in SH-SY5Y cells after 24 h of VZV infection. **(B)** Volcanic map showed the number and distribution of DEGs in SH-SY5Y cells after 48 h of VZV infection. Volcano map showed that VZV group had significant mRNA expression compared with mock without VZV infection. Log_2_ (Fold change) and log_10_ (*Q-*value) of DEGs are expressed as abscissa and ordinate, respectively. The significantly upregulated genes are shown in red and the significantly downregulated genes are shown in green.

To clarify the molecular mechanism of post-herpes neuralgia induced by VZV, we used VZV to infect SH-SY5Y cells to map the transcriptome changes of VZV-infected nerve cells. Transcriptome data were generated by RNA-Seq after infection of SH-SY5Y cells with VZV for 24 and 48 h, respectively. Quantitative expression of all genes was obtained by data quality control, filtering, and mapping, and differential expression genes were generated by deseq2 and edge algorithm. The purpose of obtaining DEGs is to reveal the differences between samples at the level of gene transcription. Figure 2 shows the representative distribution of upregulated or downregulated genes in SH-SY5Y cells after VZV infection. Our data showed that after 24 h of VZV infection, 1,373 genes were upregulated and 1,000 genes were downregulated in SH-SY5Y cells (Figure 2A). After 48 h of virus infection, 1,243 genes were upregulated and 1,661 genes were downregulated in SH-SY5Y cells (Figure 2B).

**FIGURE 3:**
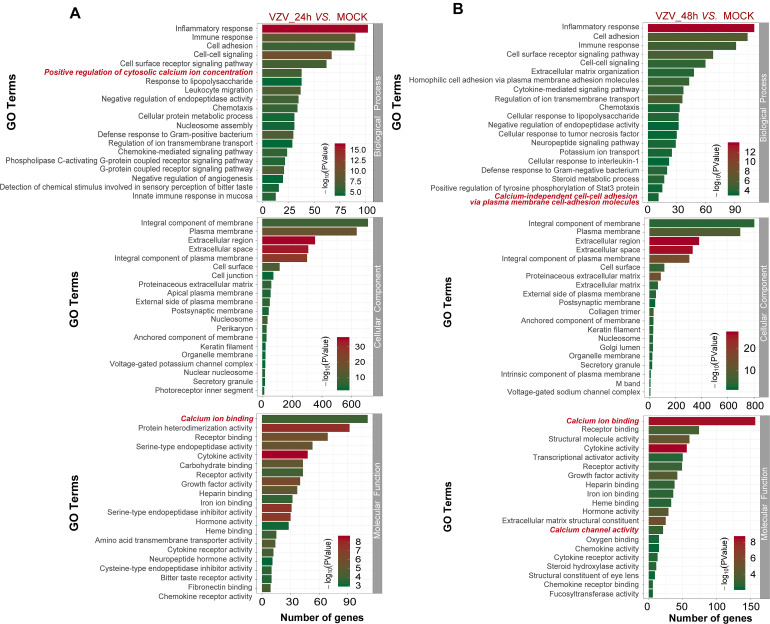
The functional enrichment of DEGs was analyzed by gene ontology classification. **(A)** GO analysis of DEGs in SH-SY5Y cells 24 h after VZV infection. **(B)** GO analysis of DEGs in SH-SY5Y cells 48 h after VZV infection. The number of DEGs was plotted as abscissa and GO terms were plotted as ordinate. It shows the top 20 highly representative GO terms enriched in DEGs, including biological processes, cellular components, and molecular functions.

To better explore the related functions of DEGs in SH-SY5Y cells infected with VZV, the GO analysis was used to enrich and classify DEGs (Figure 3). Figure 3 shows the top 20 highly representative GO terms rich in DEG, which contributes to understanding the response of SH-SY5Y cells to VZV. GO analysis clarified the top 20 biological processes of DEGs, including “inflammatory response,” “immune response,” “response to lipopolysaccharide,” “chemotaxis,” “positive regulation of cytosolic calcium ion concentration,” and “calcium-independent cell–cell adhesion via plasma membrane cell-adhesion molecules,” indicating that VZV infection caused strong inflammatory and immune responses in SH-SY5Y cells, and calcium ions might be involved in the pathogenicity of the virus. It identified and enriched the top 20 cellular component terms associated with “extracellular region,” “cell junction,” “postsynaptic membrane,” “secret granule,” and “voltage gated potential channel complex,” indicating that the response to VZV in SH-SY5Y cells involved sensory neuron signal transduction and intercellular communication. Moreover, enriched molecular functions were defined to be associated with “calcium ion binding,” “receptor binding,” “cytokine activity,” “receptor activity,” and “calcium channel activity,” implying that cell signal transduction and calcium ion transport were induced in SH-SY5Y cells by VZV infection. In conclusion, GO analysis revealed that inflammatory response, immune response, signal transduction, and calcium channel transport activities were mainly the response of SH-SY5Y cells to VZV infection.

### KEGG Analysis of DEGs in SH-SY5Y Cells Infected With VZV

**FIGURE 4:**
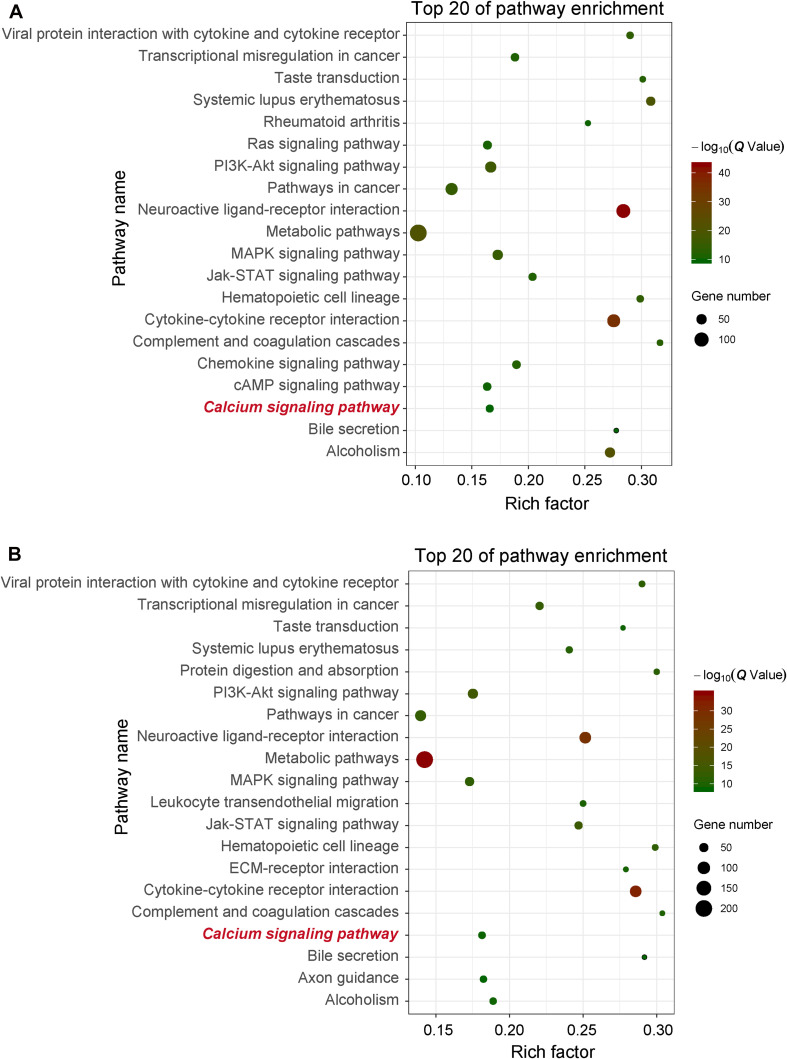
KEGG enrichment of DEGs induced by VZV in SH-SY5Y cells. **(A)** The DEGs enriched KEGG pathway was induced by VZV infection in SH-SY5Y cells for 24 h. **(B)** The DEGs enriched KEGG pathway was induced by VZV infection in SH-SY5Y cells for 48 h. The graph shows the top 20 significantly enriched KEGG pathways by plotting rich factors as abscissa and KEGG terms as ordinates.

**FIGURE 5:**
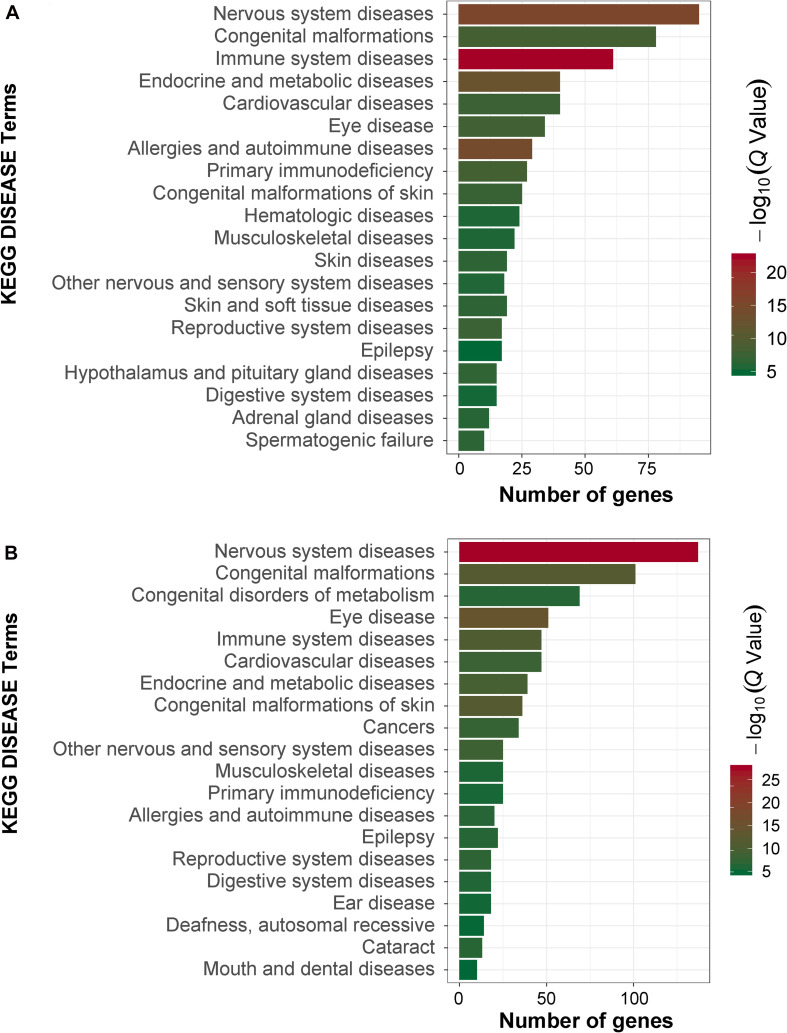
KEGG disease analysis of DEGs in SH-SY5Y cells induced by VZV. **(A)** KEGG disease analysis of DEGs induced by VZV infection of SH-SY5Y cells for 24 h. **(B)** KEGG disease analysis of DEGs induced by VZV infection of SH-SY5Y cells for 48 h. The graph shows the first 20 significantly abundant KEGG diseases by using the number of differentially expressed genes as the abscissa and the KEGG term as the ordinate.

In order to obtain signal pathways and disease classifications enriched by DEGs after VZV infection in SH-SY5Y cells, we used the KOBAS 3.0 online database to annotate the host DEGs produced by the virus. The DEGs produced by VZV are significantly enriched in “neuroactive ligand–receptor interaction,” “cytokine–cytokine receptor interaction,” “chemokine signaling pathway,” and “calcium signaling pathway” (Figure 4). These results indicated that VZV infection causes significant signal transduction and calcium signal activity in SH-SY5Y cells. In addition, we further characterized the DEGs through the KEGG DISEASE database. As shown in Figure 5, the results of KEGG DISEASE showed that DEGs were mainly enriched in “nervous system diseases,” “immune system diseases,” “skin diseases,” and “other nervous and sensory system diseases.” The results showed that the DEGs induced by VZV in SH-SY5Y cells were mainly involved in neurological and sensory diseases. The above results further confirmed that signal transduction, immune response, and calcium signal activity were involved in neurological and sensory diseases induced by VZV.

### Transcriptome Comparison Between SH-SY5Y Cells Infected With VZV and DRG in Rats With VZV-Induced PHN

Neuronal damage caused by VZV infection is generally considered to be related to the formation of neuralgia after HZ. Nerve damage is usually accompanied by dysfunction of ion channels. The dysfunction of ion channels causes abnormal ion signals in cells, which will be the cause of pain and persistence. Compared with the mock group without virus infection, our RNA-Seq results showed that the DEGs produced by VZV-infected neuroblastoma cells significantly enriched the biological processes related to calcium channels and calcium signals (Figures 3, 4). To further clarify the role of calcium signals in PHN, as previously reported, we analyzed the gene expression microarray data in the L4–L5 dorsal root ganglia of rats with herpetic neuralgia induced by VZV. As shown in Supplementary Figure 1, GO analysis showed that compared with the control group, the DEGs in DRG of herpetic neuralgia rats were mainly enriched in “cellular calcium ion homeostasis,” “positive regulation of cytosolic calcium ion concentration,” “biological process,” “voltage gated calcium channel complex” cell component, and “calcium ion binding” molecular function. Consistent with the results of GO enrichment, KEGG signaling pathway analysis also showed that the DEGs in DRG of herpetic neuralgia rats were mainly enriched in “calcium signaling pathway” (Supplementary Figure 2). These results unanimously indicated that the calcium disorder induced by VZV infection may be involved in the occurrence and development of herpetic neuralgia.

**FIGURE 6:**
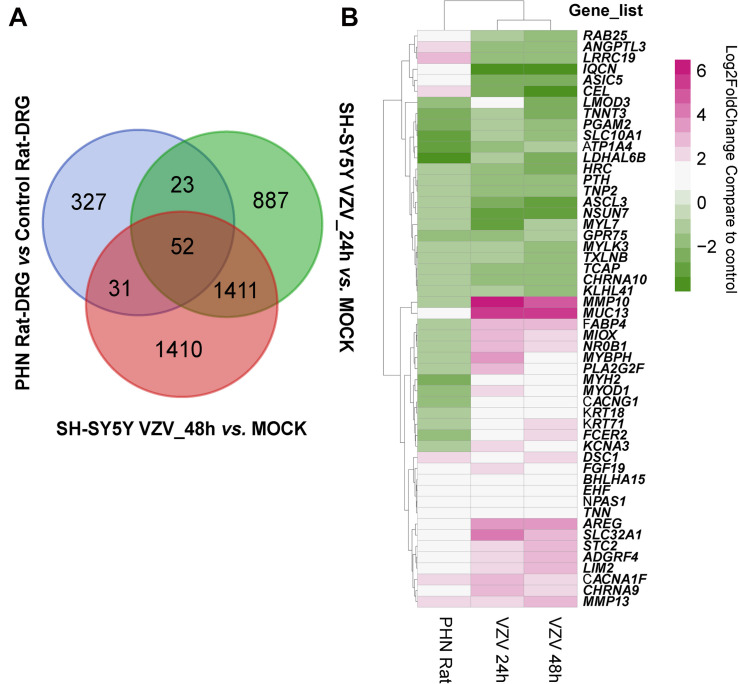
Analysis of the identical DEGs in VZV-infected SH-SY5Y cells and VZV-induced PHN rat DRG. **(A)** The Draw Venn Diagram online tool was used to obtain the identical DEGs in VZV-infected SH-SY5Y cells and VZV-induced PHN rat DRG. **(B)** The Pheatmap package was used to map the gene expression heatmap of the identical DEGs in the DRG of VZV-induced PHN rats and SH-SY5Y cells infected by VZV.

Next, we used the Draw Venn Diagram online tool to obtain a total of 52 identical DEGs in VZV-infected SH-SY5Y cells and VZV-induced DRG in herpetic neuralgia rats (Figure 6A). In addition, we used a heatmap to show the expression levels of these 52 identical DEGs based on the normalized expression amount (FPKM) of each gene (Figure 6B). Interestingly, after GO enrichment and KEGG signal analysis of these 52 identical DEGs, these identical DEGs were also closely related to calcium signaling (Table 2). In conclusion, these results further indicated that calcium signaling might play a significant role in post-herpes neuralgia induced by VZV.

**TABLE 2:** GO enrichment and KEGG pathway analysis of the same DEGs in VZV infected SH-SY5Y cells and VZV induced PHN rats DRG.

Category	Term	Genes
**Biological process**	Muscle contraction	*MYH2, MYL7, HRC, CACNG1, LMOD3*
	Sarcomere organization	*TCAP, KLHL41, MYLK3*
	**Calcium ion transmembrane transport**	***CHRNA9, CHRNA10, CACNG1, CACNA1F***
	Muscle filament sliding	*MYH2, TNNT3, TCAP*
	Regulation of striated muscle contraction	*TNNT3, MYBPH*
	Cardiac myofibril assembly	*TCAP, MYLK3*
	Myofibril assembly	*KLHL41, LMOD3*
	Detection of mechanical stimulus involved in sensory perception of sound	*CHRNA9, CHRNA10*
**Cellular component**	A band	*MYH2, MYL7*
	Myosin filament	*MYH2, MYBPH*
	Pseudopodium	*RAB25, KLHL41*
	M band	*KLHL41, LMOD3*
	Acetylcholine-gated channel complex	*CHRNA9, CHRNA10*
	Extracellular space	*MMP13, PTH, STC2, ANGPTL3, MUC13, CEL, AREG, MMP10*
	Myofibril	*MYH2, MYOD1*
	**Voltage-gated calcium channel complex**	***CACNG1, CACNA1F***
**Molecular function**	Integrin binding	*FCER2, TNN, ANGPTL3*
	Tropomyosin binding	*TNNT3, LMOD3*
	**Calcium ion binding**	***PLA2G2F, MYL7, MMP13, HRC, DSC1, MMP10***
	Acetylcholine-activated cation-selective channel activity	*CHRNA9, CHRNA10*
**KEGG pathway**	Cardiac muscle contraction	*CACNG1, ATP1A4, CACNA1F, HRC*
	Oxytocin signaling pathway	*CACNG1, MYLK3, CACNA1F, DSC1*
	Pancreatic secretion	*ATP1A4, CEL, PLA2G2F*
	MAPK signaling pathway	*CACNG1, FGF19, CACNA1F, AREG*
	Adrenergic signaling in cardiomyocytes	*CACNG1, ATP1A4, CACNA1F*
	cGMP-PKG signaling pathway	*ATP1A4, MYLK3, CACNA1F*
	**Calcium signaling pathway**	***CACNA1F, MYLK3, HRC***
	Focal adhesion	*MYL7, TNN, MYLK3*
	**Endocrine and other factor-regulated calcium reabsorption**	***ATP1A4, PTH***

*Bold values represents calcium related items and genes.*

### VZV Infection Causes Abnormal Expression of Calcium-Related Genes in SH-SY5Y Cells

**FIGURE 7:**
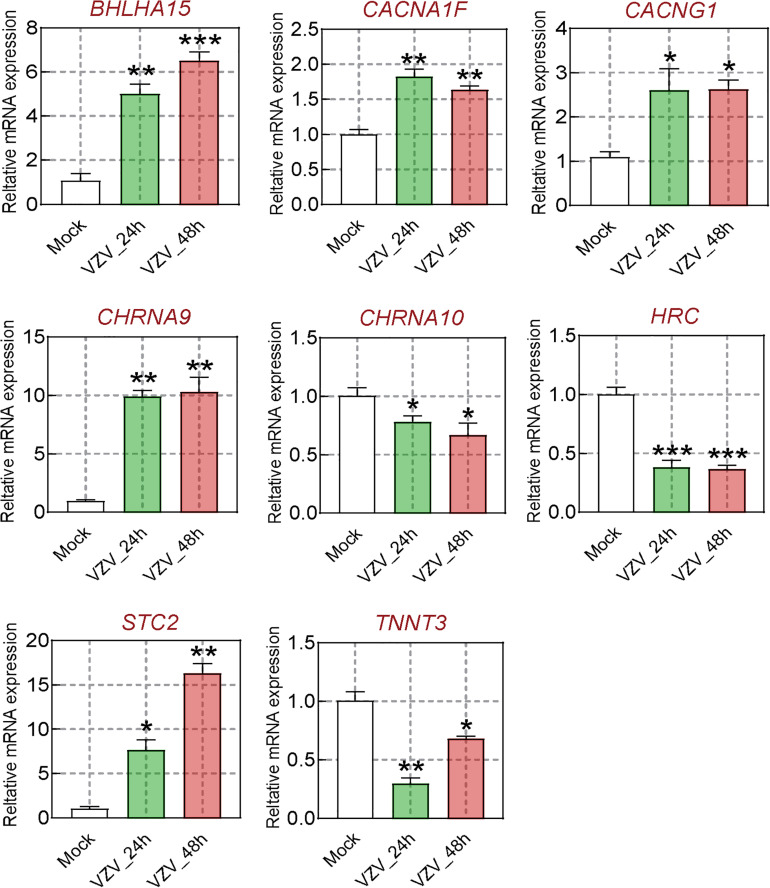
The calcium signal DEGs were verified by qRT-PCR. After VZV-infected SH-SY5Y cells for 24 and 48 h, respectively, qRT-PCR was used to quantify the expression levels of *BHLHA15*, *CACNA1F*, *CACNG1*, *CHRNA9*, *CHRNA10, HRC*, *STC2*, and *TNNT3* genes. Data are expressed as the mean ± SD from at least three independent experiments (compared to the Mock group, *P < 0.05, **P < 0.01, ***P < 0.001).

We obtained the gene expression profile of SH-SY5Y cells after VZV infection by RNA-Seq sequencing and compared with the DRG gene chip data of PHN rats induced by VZV. These data all indicated that calcium signaling may play an important role in VZV-induced PHN. In Figure 6, we have obtained 52 identical DEGs by VZV-infected SH-SY5Y cells and DRG of PHN rats. Using the GeneCards (Fishilevich et al., 2016) online database^5^ to annotate these shared DEGs, we got the genes related to calcium signaling as *BHLHA15*, *CACNA1F*, *CACNG1*, *CHRNA9*, *CHRNA10*, *HRC*, *STC2*, and *TNNT3*. We examined the mRNA expression levels of these genes in SH-SY5Y cells 24 h and 48 h after VZV infection. Our results showed that VZV infection of SH-SY5Y cells significantly upregulated the expression of *BHLHA15*, *CACNA1F*, *CACNG1*, *CHRNA9*, and *STC2*, but significantly downregulated the expression of *CHRNA10*, *HRC*, and *TNNT3* (Figure 7). These results confirmed the validity of our transcriptome data and suggested that VZV might mediate calcium disorder in SH-SY5Y cells by disrupting the expression of calcium-related proteins. However, the expression patterns of these genes were different in VZV-infected SH-SY5Y cells, suggesting that these genes might be involved in VZV-induced PHN through different mechanisms.

### VZV Infection Causes Calcium Signal Disorder in ARPE-19 and SH-SY5Y Cells

**FIGURE 8:**
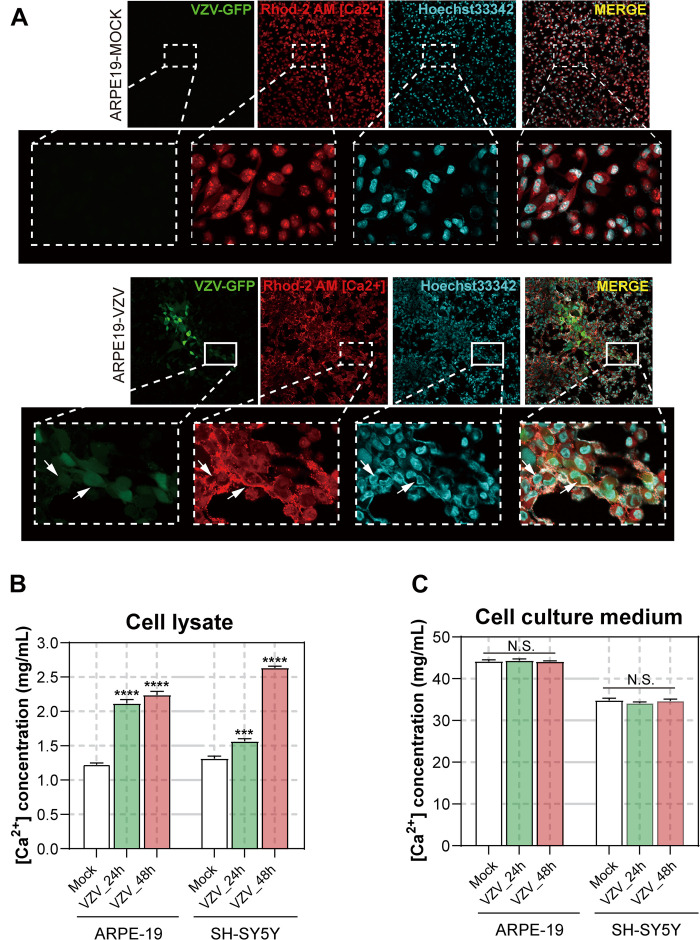
Effect of VZV on Ca^2+^ in ARPE-19 and SH-SY5Y cells. **(A)** The Rhod-2 AM calcium ion probe was used to assess the intracellular calcium ion distribution of ARPE-19 cells 72 h after VZV infection. The calcium colorimetric assay kit was used to evaluate the calcium ion content in the cell lysate **(B)** and cell culture supernatant **(C)** after VZV infection of ARPE-19 and SH-SY5Y cells at 24 h and 48 h, respectively. Data are expressed as the mean ± SD from at least three independent experiments (compared to the Mock group, ***P < 0.001, ****P < 0.0001, N.S.; not significant). The white arrows indicate the distribution of calcium ions on the cell membrane surface.

In order to obtain further visual evidence that VZV causes calcium disorder in infected cells, we directly assessed the distribution and content of calcium ions in ARPE-19 and SH-SY5Y cells infected with VZV. After 72 h of infecting ARPE-19 cells with VZV, we used Rhod-2 AM calcium ion probe to examine the effect of the virus on the distribution of calcium ions in the infected cells. As shown in Figure 8A, in ARPE-19 cells without VZV infection, Ca^2+^ was evenly filled into the whole cell (Figure 8A, upper part). After infection with VZV for 72 h in ARPE-19 cells, the VZV recombinant strain carrying green fluorescent protein could be clearly observed in ARPE-19 cells (Figure 8A, bottom, left). According to the fluorescence images of the nuclear dye Hoechst 33342, we observed that the virus caused ARPE-19 cells to form a significant multi-nucleus syncytial disease state (Figure 8A, bottom, right). Interestingly, we found strong Ca^2+^ signals around the cell membrane in VZV-infected cells (Figure 8A, bottom, center). These results indicated that VZV might cause intracellular calcium disorder by affecting the distribution of calcium ions in cells.

To clarify the effect of VZV infection on the calcium content in cells, we used the/a calcium colorimetric assay to evaluate the calcium content in cell lysates and cell culture supernatants. The results are shown in Figures 8B,C; compared with the mock group, VZV infection significantly increased the calcium content in infected cells (Figure 8B, *P* < 0.001), while the calcium ion content in cell culture supernatants did not change significantly before and after the virus infection.

## Discussion

The pathogenesis of herpetic neuralgia is complex and unclear, leading to the poor clinical treatment of herpetic neuralgia, especially PHN. In this study, transcriptome sequencing was used to obtain a transcriptional map of neuroblastoma cells acutely infected with VZV. We tried to reveal the response of nerve cells to VZV infection from the transcription map and explore the evidence that may be related to herpetic neuralgia. Neuroblastoma cells infected with VZV for 48 h showed obvious cytopathic states such as cell rounding, swelling, and syncytial formation (Figure 1A). Transcriptome sequencing results demonstrated that thousands of host DEGs were produced in this neurocytopathic state induced by VZV (Figure 2). The large number of DEGs induced by VZV also confirmed that VZV in humans would cause great damage to nerve cells from incubation to activation of infection.

The activation and infection of VZV in sensory neurons will cause severe inflammation and immune response, which is the body’s defense response to pathogenic microorganisms (Almanzar et al., 2019; Cote-Daigneault et al., 2019). Similarly, our GO enrichment results showed that a large number of VZV-induced DEGs in neuroblastoma cells were mainly related to “inflammatory response,” “immune response,” “response to lipopolysaccharide,” and “chemotaxis” (Figure 3). These results indicated that neuroblastoma cells strongly respond to VZV infection. Additionally, we further analyzed and found that the GO enrichment results of these DEGs induced by VZV pointed to “positive regulation of cytosolic calcium ion concentration,” “voltage-gated potential channel complex,” “calcium ion binding,” and “calcium channel activity,” suggesting that the intracellular calcium disorder caused by virus might be related to the pathogenicity of virus. KEGG signaling pathway enrichment analysis revealed that the DEGs produced by VZV were mainly enriched in “neuroactive ligand receptor interaction,” “cytokine–cytokine receptor interaction,” “chemokine signaling pathway,” and “calcium signaling pathway” (Figure 4). Consistently, our transcriptome data suggested that calcium signaling disorders play an important role in VZV-induced neuroblastoma cell lesions. Previous reports have shown that VZV infection induced sensitivity to adrenergic stimulation in cultured nociceptive DRG neurons, causing Ca^2+^ levels to increase after stimulation with norepinephrine or adrenergic agonists (Schmidt et al., 2003). The increase in Ca^2+^ is considered to be consistent with pain and hyperalgesia and is also involved in heat sensitization and thermal hyperalgesia of nociceptors (Kress and Guenther, 1999). One of the typical symptoms of HZ pain and PHN is hyperalgesia and thermal hyperalgesia, which may be related to VZV-induced adrenergic stimulation sensitivity and change of calcium signal transduction in nerve cells (Schmidt et al., 2003; Eyigor et al., 2006).

We tried to find evidence that calcium disorder could be involved in the pathogenesis of PHN in rats with HZ neuralgia. We analyzed the previously reported GSE64345 data set, which included microarray profiling from the ipsilateral L4–L5 DRG of PHN rats (Guedon et al., 2015). The results of GO enrichment showed that DEGs from the PHN rat model were mainly concentrated in the biological process of “cellular calcium ion homeostasis,” “positive regulation of cytosolic calcium ion concentration,” cell component of “voltage gated calcium channel complex,” and molecular function of “calcium ion binding” (Supplementary Figure 1). Consistent with previous reports, KEGG signaling pathway analysis showed that DEGs in DRG of the PHN rat model were mainly enriched in “calcium signaling pathway,” “neuroactive ligand-receptor interaction,” and “serotonergic synapse” (Supplementary Figure 2; Qiu et al., 2020). Encouragingly, our results further revealed that the GO enrichment and KEGG signaling pathway analysis of 52 identical DEGs (Figure 6) obtained from the VZV-infected SH-SY5Y cells and the VZV-induced PHN rat DRG all showed calcium signaling-related items (Table 2). Many voltage-gated and ligand-gated Ca^2+^ channels are expressed in neurons, which play an important role in the regulation of Ca^2+^ influx in neurons and glia of central nervous system (CNS) (Mei et al., 2018). At the same time, there has been more and more evidence that Ca^2+^ signals are involved in the process of pain and other central nervous system diseases (Gemes et al., 2011; Mei et al., 2018). Therefore, all these results suggest that calcium signaling may play a key role in the progression of PHN disease. These could be attributed to the fact that VZV induces abnormal voltage-gated and ligand-gated Ca^2+^ channel expression in neurons to drive intracellular calcium disorders, thereby maintaining the persistence of pain.

It has already been reported that *BHLHA15*, *CACNA1F*, *CACNG1*, *CHRNA9*, *CHRNA10*, *STC2*, *HRC*, and *TNNT3* participated in calcium ion transport and/or mediate intracellular calcium signals (Burgess et al., 2001; Luo et al., 2005; Zeiger et al., 2011; An et al., 2012; Fasciani et al., 2013; Del Bufalo et al., 2014; Wei and Jin, 2016; Romero et al., 2017). As a member of the basic helix loop helix (bHLH) protein family, *BHLHA15* (also known as *MIST1*) knockout will lead to sustained high levels of cytoplasmic Ca^2+^ in acinar cells and affect the secretion function of cells (Luo et al., 2005; Garside et al., 2010). *CACNA1F* encodes the L-type calcium channel alpha 1 subunit Ca_v_1.4 protein, which was previously shown to significantly increase its mechanical threshold latency after mutation in rats (An et al., 2012). Ca^2+^ channel γ (*CACNG1*) subunit is a membrane protein, which has been proved to have multiple homologous genes in different tissues and involved in calcium transport (Burgess et al., 1999, 2001). Cholinergic receptor nicotinic alpha 9/10 (*CHRNA9*/*CHRNA* 10, also known as α9α10 nAChR) belongs to the ligand gated ion channel family, which is related to the process of a variety of neuropathic pain (e.g., neuropathy pain, nerve injury, and diabetes pain) (Romero et al., 2017). Stanniocalcin 1 (STC1) proteins have been widely proposed to be regulators of Ca^2+^ homeostasis, and it has been shown to be a negative regulator of store-operated Ca^2+^ entry (SOCE) to regulate intracellular Ca^2+^ levels (Zeiger et al., 2011). Histidine-rich calcium-binding protein (HRC) is a high-capacity, low-affinity calcium-binding protein, which has been confirmed to be involved in the regulation of sarcoplasmic reticulum (SR) release of Ca^2+^ (Kim et al., 2003). Troponin T type 3 (*TNNT3*) encodes the fast skeletal muscle isoform of troponin T (fsTnT), which is required for Ca^2+^-mediated activation of actomyosin ATPase activity.

Generally, the abnormal expression of many calcium signal-related genes induced by VZV is bound to affect the intracellular calcium homeostasis of infected cells. As shown in Figure 8, we directly measured the distribution and content of calcium ions in ARPE-19 and SH-SY5Y cells infected with VZV and found that the virus could change the distribution of calcium ions in infected cells and significantly increase the intracellular calcium content. In fact, the first-line drugs such as gabapentin and pregabalin for the treatment of PHN are calcium channel modulators, which can inhibit hyperalgesia and central sensitization by binding to the α2–δ subunit of voltage-gated calcium channel (VGCC) (McKeage and Keam, 2009; Ifuku et al., 2011; Gudin et al., 2019). However, gabapentin and pregabalin can effectively relieve pain to a certain extent and reduce the occurrence of neuralgia after HZ, but they still cannot fundamentally prevent the occurrence of PHN (Mahn and Baron, 2010). We speculated that activated VZV infection in sensory ganglia could cause abnormal expression of various types of calcium channel proteins. Gabapentin and pregabalin may only regulate one type of calcium channel, but cannot block the intracellular calcium disorder caused by other abnormally expressed calcium channels.

## Conclusion

Altogether, our results showed that VZV not only changed the expression pattern of calcium signal-related proteins but also disrupted the intracellular calcium ion distribution and calcium ion content, ultimately leading to calcium disorders in infected cells. These findings are unique in VZV infection, which may help to understand the pathogenesis of PHN better. Practically, the persistence of this calcium disorder in nerve cells may become an important cause of PHN and serves as a potential target for clinical diagnosis and treatment of PHN.

## Data Availability Statement

Publicly available datasets were analyzed in this study. This data can be found here: Sequencing data set supporting the results of this article has been submitted to the NCBI Gene Expression Omnibus (GEO) database, and the accession number is GSE141932.

## Ethics Statement

This study was approved by the Ethics Committee of Shenzhen Nanshan People’s Hospital and The 6th Affiliated Hospital of Shenzhen University Health Science Center.

## Author Contributions

SW, DX, WS, and LX supervised and coordinated the study and designed the experiments. SW and MO conducted and performed all experiments. SY, MO, JC, and JH assisted various portions of the experiments and analysis of data. SW, SY, MO, and JC wrote the manuscript. All authors contributed to the article and approved the submitted version.

## Conflict of Interest

The authors declare that the research was conducted in the absence of any commercial or financial relationships that could be construed as a potential conflict of interest.

## Footnotes

1 http://cran.r-project.org/web/packages/gplots/index.html

2 http://bioinformatics.psb.ugent.be/webtools/Venn/

3 https://david.ncifcrf.gov/

4 http://kobas.cbi.pku.edu.cn/kobas3

5 https://www.genecards.org/
