# Supplementary material for: Transcriptome Analysis Reveals the Role of Cellular Calcium Disorder in Varicella Zoster Virus-Induced Post-Herpetic Neuralgia
Source: Front Mol Neurosci. 2021 May 17;14:665931. doi: 10.3389/fnmol.2021.665931 (PMC8166323; doi:10.3389/fnmol.2021.665931)
Supplement: Supplementary file 1 [file Table_1.DOCX]

**Supplementary material**


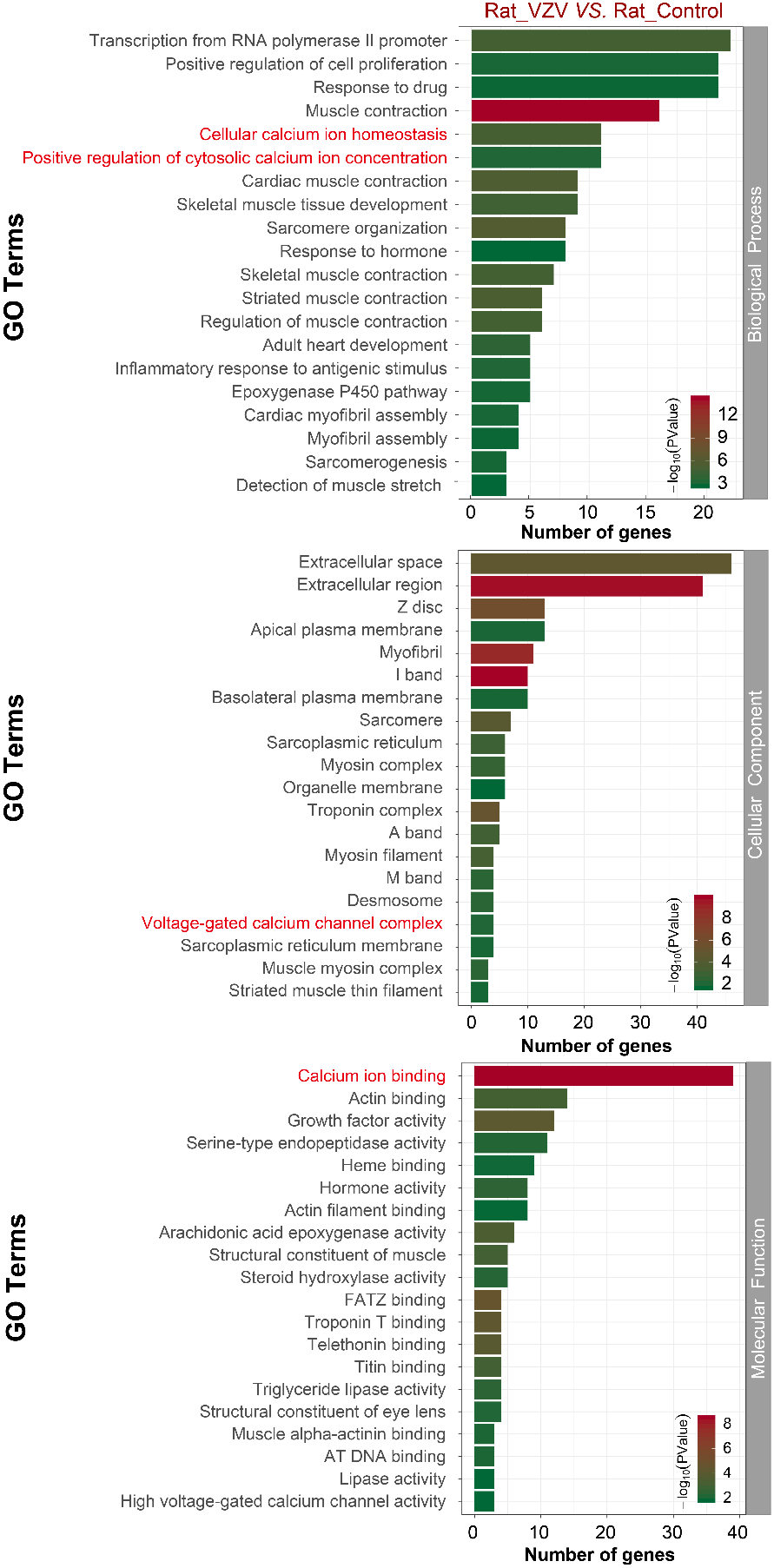


**Fig. S1** Gene ontology analysis of differentially expressed genes in dorsal root ganglia of PHN rats. The number of DEGs was plotted as abscissa and go terms as ordinate. It shows the top 20 highly representative GO terms enriched in DEGs, including biological processes, cellular components and molecular functions.

**
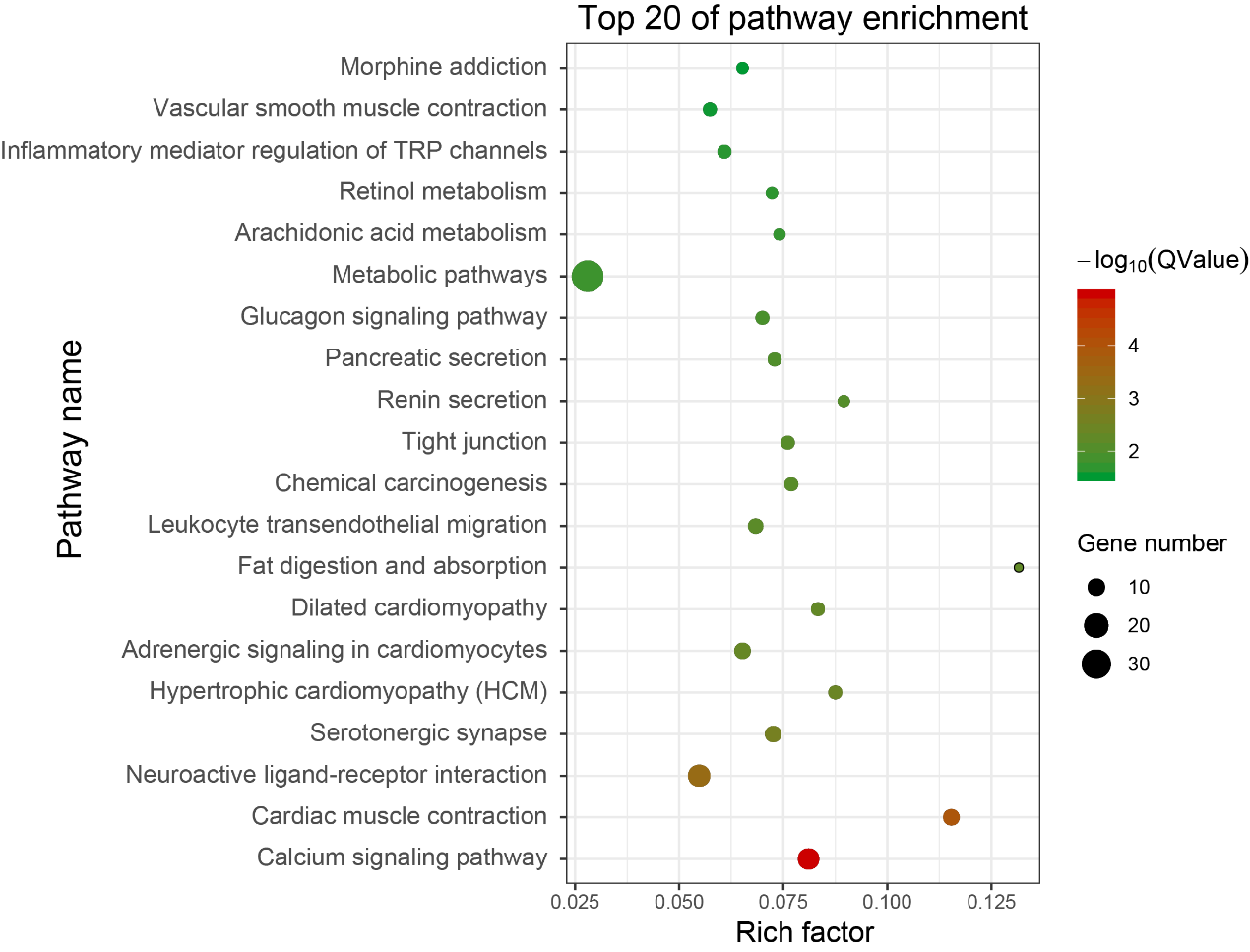
**

**Fig. S2** KEGG signaling pathway analysis of differentially expressed genes in dorsal root ganglia of PHN rats. The graph shows the top 20 significantly enriched KEGG pathways by plotting rich factors as abscissa and KEGG terms as ordinates.
